# Qualitative Aspects of Bone Marrow Adiposity in Osteoporosis

**DOI:** 10.3389/fendo.2016.00139

**Published:** 2016-10-25

**Authors:** Ana María Pino, Melissa Miranda, Carolina Figueroa, Juan Pablo Rodríguez, Clifford J. Rosen

**Affiliations:** ^1^Laboratory of Cell Biology, INTA, University of Chile, Santiago, Chile; ^2^Maine Medical Center, Portland, ME, USA

**Keywords:** lipids, fatty acids, unsaturated, bone marrow cells, bone marrow examination, adipocytes

## Abstract

The function of marrow adipocytes and their origin has not been defined although considerable research has centered on their presence in certain conditions, such as osteoporosis. Less work has focused on the qualitative aspects of marrow fat. Bone marrow serum is composed of multiple nutrients that almost certainly relate to functional aspects of the niche. Previous studies using non-invasive techniques have shown that osteoporotic individuals have more marrow fat and that the ratio of saturated: unsaturated fatty acid is high. We recently reported that bone marrow sera from osteoporotic patients with fracture showed a switch toward decreased content of total saturated versus unsaturated fatty acids, compared to patients without fracture highlighting a dynamic relationship between the composition of fatty acids in the bone microenvironment and the metabolic requirements of cells. The relative distribution of fatty acids differed considerably from that in the serum providing further evidence that energy utilization is high and that marrow adipocytes may contribute to this pool. Whether these lipids can affect osteoblast function in a positive or negative manner is still not certain but will require further investigation.

## The Bone Marrow Microenviroment

In adults, the rigid bone structure of the cortex encloses a dynamic and flexible cell organization sustaining continuous bone and bone marrow stroma remodeling, as well as blood formation. These processes rest on preserving specific adult stem cells (SCs) that are characterized by their capacity for self-renewal and multilineage differentiation. These unique properties of SCs are not only cell-autonomous *in vivo*, but also controlled by their surrounding microenvironment, which is currently called the SC niche. The original Schofield’s proposition for hematopoietic stem cells (HSCs) in the bone marrow emphasized the input from other marrow cells types to maintain SCs behavior and prevent maturation (1). Such cell- depending interactions provide a specialized microenvironment (“niche”) that allows cell lodging while maintaining self-renewal of SCs; loss of such an association would lead to cell differentiation. At present, the niche concept has been assumed to explain the behavior of SCs in several tissue types while evolving to take account of specific cell types, anatomical sites, soluble molecules, signaling cascades and gradients, as well as physical factors, such as weight bearing, shear stress, oxygen tension, and temperature (2–5). Among the most studied somatic SCs in mammals are the HSC and mesenchymal stromal/stem cells (MSCs) that integrate a unique niche in the bone marrow made of heterotypic SC pairs (6). Therefore, the bone marrow niche concept comprises a complex microenvironment, providing spatial and temporal coordinated signals to support SC function; balanced inputs from the niche can sustain homeostatic SC self-renewal and differentiation, but under pathological conditions they could constrain SCs functioning.

## Mesenchymal Stem Cells or Marrow Stromal Cells

In the bone marrow, MSCs mainly localize lining blood vessels, in particular on the sinusoids, the characteristic vessels of bone marrow microcirculation (7, 8), often in close association with HSCs (6, 9, 10). Then, the sinusoidal wall brings together both HSCs and MSCs, framing and sustaining a mutual niche (6, 11). Such perivascular localization has also been demonstrated for MSCs in several tissues through the body, which support the proposition that MSCs are fundamental to the healing of many tissues. MSCs in the bone marrow are ontogenically distinct populations taking over various biological functions, such as niches for HSCs (6, 12, 13), progenitor cells for bone formation during bone remodeling or repair (14), for cartilage and adipocyte formation, and vascular support (15–17).

*Ex vivo*, hMSCs are relatively easy to obtain from a small bone marrow aspirate and they can simply be isolated from other marrow nucleated cells by their adherence to plastic dishes. Subsequently, these cells can be expanded under *in vitro* conditions in which they display varying proliferation and differentiation potential; however, as cells are expanded consecutively under standard conditions, they lose some of these capacities. Clonal analysis demonstrated that MSCs obtained from the bone marrow are a heterogeneous mixture of cells that differ in their stage of lineage commitment and extent of differentiation (18–21). Therefore, MSCs populations in the bone marrow or those that are isolated and maintained in culture are not homogeneous, but rather assemble progenitor cells with diverse biological properties, such that not all MSCs are alike.

In theory, the lineage fate of MSCs appears to be determined during cell “commitment,” at very early stages of cell differentiation. During this almost unknown period, both intrinsic (genetic) and environmental (local and/or systemic) conditions interplay to outline the cell’s fate toward one of the possible lineages. Factors, such as age (22), culture condition (23), microenvironment (24), mechanical strain (25), and some pathologies (26, 27), appear to affect the intrinsic activity of MSCs.

Multilineage differentiation capacity has been related to wide range gene expression at intermediary levels, which along cell commitment and differentiation shifts to a selective mode of restricted genes expressed at high level (21, 28–30). Several fundamental signaling pathways participate in regulating the lineage commitment of MSCs, including transforming growth factor-beta (TGFβ)/bone morphogenic protein (BMP) signaling, wingless type MMTV integration site (Wnt) signaling, Hedgehogs (Hh), Notch, and fibroblast growth factors (FGFs) (31, 32).

## Relationship Between the Osteo/Adipogenic Processes the Obesity Theory of Osteoporosis

The formation, maintenance, and repair of bone tissue depend on fine-tuned interlinks in the activities of cells derived from the two SC types housed in the bone marrow interstice, above described. HSCs along the myeloid differentiation lineage generate osteoclasts (33), whereas osteoblasts derive from MSCs, which are also progenitor cells for adipocytes (34). Bone diseases, such as osteopetrosis, osteopenia, and osteoporosis, show imbalance between bone formation and resorption (33).

Since MSCs can generate several cell types, the control of lineage commitment of MSCs appears critical. Alteration in such control has been observed in some bone diseases resulting in abnormal bone remodeling, which show divergent commitment of MSCs to adipocytes and osteoblasts. For instance, increased marrow fat content has been demonstrated in osteoporosis patients, the most common bone remodeling disorder (35, 36). Also, this alteration of the osteo/adipogenic processes is observed in other bone loss conditions, such as aging, immobilization, microgravity, ovariectomy, diabetes, and glucocorticoid or thiazolidinedione treatments, highlighting the harmful consequence of marrow adipogenesis in osteogenic disorders (37–39).

Osteoblasts and adipocytes originate from a common precursor, MSCs; preserving bone tissue requires adequate osteoblastic differentiation while minimizing adipogenesis. Commitment and differentiation of MSCs into a specific phenotype *in vivo* is postulated to be controlled by hormonal and local factors (paracrine/autocrine) regulating the expression and/or activity of master differentiation genes (32, 40). Research on MSCs differentiation *in vitro* showed that activation of PPARγ2 and C/EBPs match to the master transcription factors for adipogenic differentiation (41–43), while Runx2 and osterix are required for osteogenic differentiation (44). PPARγ2 positively regulates adipocyte differentiation while acting as a dominant negative regulator of osteogenic differentiation (45, 46). By contrast, an increase in bone mass density was observed in PPARγ2-deficient mice model (47). On the other hand, Runx2 expression by MSCs inhibits their differentiation into adipocytes, as shown by experiments in Runx2/calvarial cells, which spontaneously differentiate into adipocytes (48). However, due to the heterogeneity of the earliest MSC, cell expression of only adipogenic or osteogenic markers is unlikely. Indeed, osterix positive marrow adipocytes have been shown in lineage tracing studies, and marrow adipocytes can be traced to peroxiredoxin 1 (Rosen, personal communication). Moreover, some Runx2-positive cells isolated in high marrow adiposity states also have large lipid droplets and express perilipin (49).

Thus, in most cases, there is a reciprocal relationship in the regulation of these differentiation processes whose alteration would facilitate adipose accretion in the bone marrow, at the expense of osteoblast formation, decreasing bone mass (50–52). Such altered conditions would prevail in the bone marrow of osteoporotic patients and other bone loss conditions, disrupting the activities of MSCs and their microenvironment (37, 40, 50). This proposition has been termed the obesity theory of osteoporosis.

According to this proposition, observations in iliac crest biopsies from elderly women show considerable accumulation of adipocytes; i.e., 70–80% of the total marrow volume in osteoporotic patients, compared to that of healthy elderly women. More recently, observations done by magnetic resonance imaging (MRI) allowed similar conclusion in patients with low bone density (53–55). However, the origin of marrow adipocytes and their true nature has been more difficult to discern.

## Human Bone Marrow Fat

In newborn mammals, there is scarce marrow fat; however, adipocyte number increases with age such that in humans older than 30 years, most of the femoral cavity is occupied by adipose tissue (56). The function of human marrow fat was largely overlooked; at first, it was considered “filler” for the void left by trabecular bone during aging or after radiation treatments. Early observations from the 1970s highlighted the different characteristics between adipocytes within the red and yellow marrow, suggesting that marrow adipocytes may have a region-specific lipid composition (57, 58). In humans, adipocytes accumulate within the yellow marrow at or slightly before birth, regardless of prematurity, and accelerate between 4 and 8 weeks of age (59, 60). Early adipose tissue is established in distal skeletal regions, including the hands, feet, and distal tibia. This is often called constitutive marrow fat. After attaining some bulk, distal marrow adipose tissue histologically resembles peripheral white adipose tissue, and is relatively devoid of active hematopoiesis. During adulthood, marrow fat accretion continues in areas of red, hematopoietic marrow (61), which show histologically single adipocytes interspersed with sites of active hematopoiesis. These regions appear to be spatially distinct in rats and mice; but in human, both types of adipose tissue may allocate in the same skeletal region (62).

In a recent study in mice, Scheller et al. (62) related region-specific fat differences in development to functional differences, such as regulation, adipocyte size, lipid composition, gene expression, and genetic determinants of marrow adipocytes, proposing that distal fat areas embody constitutive marrow adipose tissue (cMAT), while proximal adipose tissue contains regulated marrow adipose tissue (rMAT). Although cMAT showed histologic similarities with white extra-medullar adipose tissue (WAT), the lipid composition, cold regulation, and gene expression of WAT is more related to that of rMAT, concluding that lipid metabolism in WAT and rMAT adipocytes may be similar. Of note, cMAT showed increased unsaturation index compared to both, rMAT and WAT. Scheller’s study and those of others highlight that not all marrow adipocytes are equivalent, sustaining connotation for the marrow niche and its relationship to skeletal and whole-body metabolism.

Bone marrow fat has a role in systemic and local energy metabolism (63–66). When dysfunctional, it may disrupt the complex relationships in the bone marrow microenvironment, constraining functioning of both HSCs and MSCs, and thereby affecting their progeny (67, 68).

Bone marrow adipocytes have endocrine, autocrine, and paracrine effects; the activity of these cells on neighboring marrow result from the production of adipokines, steroids, cytokines, and free fatty acids (51, 64, 69–71), which could sustain or suppress the hematopoietic and osteogenic processes (10, 50, 51, 63, 64, 72).

The modification of bone marrow adipose tissue, either in size or function, may have similar consequences to that of extra medullary fat, which by unbalanced production of signaling products underlay in several human diseases, including obesity, lipodystrophy, atherogenesis, diabetes, and inflammation.

## Marrow Fat Composition and Osteoporosis

Current information on bone marrow fat points to diverse adipocytes that have development-dependent skeletal distribution, showing distinct properties, such as lipid composition, gene expression, and probably subjected to different peripheral and local regulation. Underlying the relationship between such bone marrow fat and bone function is a balance in the number and quality of adipose cells; hence, the interest in characterizing bone marrow fat and searching for the quality of human marrow fat, for instance, in a bone remodeling disorder like osteoporosis.

Skeletal and marrow cells utilize fatty acids as substrates for their energy needs (73), although the contribution of marrow adipocytes to such requirements is most unknown; as it is the role of fatty acids stored in the marrow adipocytes in bone remodeling and hematopoiesis. Moreover, the lipid composition in the interstitial compartment surrounding bone marrow cells, its physiological significance, its variation under a bone stress situation, or its relationship with systemic lipids are undefined.

Studies on the composition of bone marrow fat suggested that it could have clinical relevance, in addition to marrow fat size and volume. Such conclusion is derived from *in situ* non-invasive MRI-based analysis of bone marrow, in patients with chronic diseases, such as osteoporosis (54), type 2 diabetes mellitus (74), or diabetic and non-diabetic women with prevalent fragility fractures (75). These studies demonstrated significantly lower unsaturation of bone marrow lipids in patients than corresponding controls. Moreover, Patsch and colleagues showed that low unsaturation and high saturation levels of lipids was accentuated in diabetic patients with prevalent fractures (75).

Physiological levels of molecules in the human bone marrow milieu are practically unknown; measurement has been particularly difficult not only because of tissue seclusion, but because of the complicated anatomy and blood perfusion of bone requiring invasive bone marrow sampling. Thus, knowledge on the availability to bone marrow cells of metabolites or regulatory compounds is scarce, limited to some pathologic condition or estimated from measurements in plasma (69, 76–78). A soluble fraction, named “bone marrow serum (BMS),” obtained after spinning human bone marrow aspirates allows measurement of physiologically significant concentrations in the bone cell microenvironment, which are dissimilar from circulating plasma or whole blood (70, 79, 80).

In such fraction, we tested the hypothesis that BMS reflects the uniqueness of the marrow compartment in respect to the sequestration and utilization of fatty acids, by studying the composition of total soluble fatty acids in BMS and plasma samples from control and osteoporotic women with and without hip fracture (81). Results revealed a specific pattern of fatty acid composition in the bone marrow milieu that was characterized by higher saturated and decreased unsaturated fatty acids, compared to that of the circulation. The proportion of fatty acids in the BMS varied in the range of lipids in plasma, but its relative levels of unsaturated and saturated fatty acids compares well to those observed in the human marrow fat tissue (62, 75, 82), implying a distinct lipid content in BMS that replicates relevant fatty acids derived from the activity of adipocytes and other marrow cells, rather than those provided by blood plasma. The lipid content in BMS of all women studied suggests an active exchange of lipids between marrow cells; thus, the surplus of saturated fatty acids in BMS could result from palmitic and stearic acids supplied mainly by adipocytes, while marrow cells apparently preserve their pool of unsaturated fatty acids, except for oleic acid that is higher in BMS than in plasma (81).

By contrast, when the content of fatty acids in BMS and blood plasma was evaluated according to women’s bone mass density, no consistent differences in fatty acid composition were apparent in BMS, nor in plasma, which is analogous to former observations on the composition of bone fat tissue (53, 54, 74). Notwithstanding, there was a trend toward higher saturation and lower unsaturation in BMS fatty acids as compared to plasma. The analysis of the relative proportion of individual fatty acids suggests an active distribution of unsaturated fatty acids.

**Figure 1:**
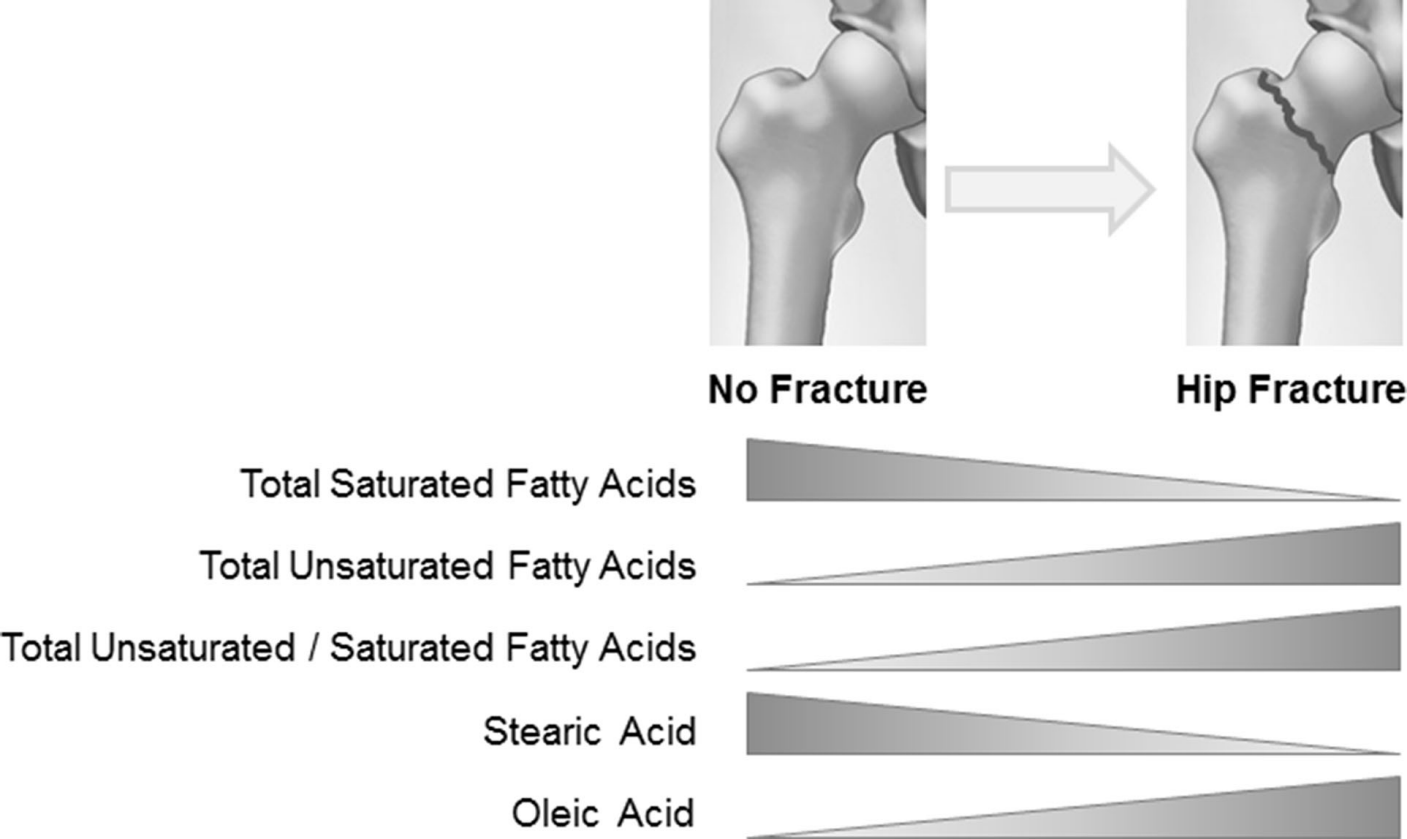
**Relationship between hip fracture and fatty acid composition in the bone marrow serum of osteoporotic women**.

The former conclusion is supported by further analysis of data on the composition of fatty acids in both BMS and plasma in the osteoporosis group, in relation to the presence or not of a hip fracture (Figure 1). A switch toward decreased content of total saturated versus unsaturated fatty acids was observed in BMS of women with fractures, emphasizing a dynamic relationship between the composition of fatty acid in the bone microenvironment and the metabolic requirements of cells. In women with fractures, stearic acid content significantly decreased concomitant with increased oleic acid content, implying a substrate to product relationship to fulfill specific requirements for unsaturated fatty acids. Moreover, increased activity of cyclooxygenase (COX), COX-2 could be proposed from the total removal of polyunsaturated eicosatrienoic and arachidonic fatty acids (81).

It is difficult to define the origin (adipocyte or other marrow cell production) and type of lipids that change the fatty acid content of the BMS fraction after fracture, in part because the measurement was limited to the total fatty acid pool in the soluble fraction of the bone marrow. After a hip fracture bone cell metabolism may change in order to provide extra energy for damage repair by osteoblasts, or to suppress excessive inflammation. Interestingly, the lipid fraction in BMS of fractured women appears adjust to such conditions, thus diminished level of saturated fatty acid could result from increased supply of energetic substrate to marrow cells (bone, fat, and hematopoietic), while an increased exchange of oleic and polyunsaturated fatty acids could be related to their regulatory functions.

In conclusion, in this report, we summarize evidence on the role that adipocytes may play in the bone marrow in health and disease. Bone marrow adipose tissue appears as distinct from other extra marrow fat tissues, heterogeneous in its development, and allocated in subpopulations that seem to have different physiological properties. The regulation of the number and quality adipocytes is a necessity to preserve a functional bone marrow microenvironment to sustain SCs. In a bone disease like osteoporosis, there is increased differentiation of the precursor MSCs toward adipocytes, altering the local marrow microenvironment but leading to bone loss. In addition to fat mass, the quality of lipids could also participate in disrupting local regulation; hence, the interest in knowing the fatty acid composition of marrow adipose tissue. The measurement of the fat composition in BMS portrays a dynamic exchange of fatty acids in this fraction implying the connotation of locally produced lipids. The fatty acid composition in the BMS is enriched in saturated fatty acids and decreased in unsaturated fatty acids, as compared to blood plasma. Therefore, it is clear that qualitative aspects of marrow adiposity provide significant insights into the metabolism of the bone marrow niche, and may offer clues as to the pathophysiology of bone diseases, such as osteoporosis.

## Ethics Statement

This study was approved by the INTA, University of Chile IRB.

## Author Contributions

JR did the research and helped prepare the manuscript. AP did the background work. CF also performed some of the experiments.

## Conflict of Interest Statement

The authors declare that the research was conducted in the absence of any commercial or financial relationships that could be construed as a potential conflict of interest.
